# Supplementary material for: Effects of Multi-Strain Probiotics and Perilla frutescens Seed Extract Supplementation Alone or Combined on Growth Performance, Antioxidant Indices, and Intestinal Health of Weaned Piglets
Source: Animals (Basel). 2022 Aug 30;12(17):2246. doi: 10.3390/ani12172246 (PMC9454523; doi:10.3390/ani12172246)
Supplement: Supplementary file 1 [file animals-12-02246-s001.zip › animals-1851690-supplementary.pdf]

Table S1. Primer sequences of RT-PCR

Gene[1]	Primer sequence (5'–3')	Size (bp) Product length	Accession no.
β-actin	Forward: TCTGGCACCACACCTTCT<br>Reverse: TGATCTGGGTCATCTTCTCAC	144	XM_021086047.1
ZO-1	Forward: CAGCCCCCGTACATGGAGA<br>Reverse: GCGCAGACGGTGTTCATAGTT	114	XM_021098896.1
IL-1β	Forward: TCTGCCCTGTACCCCAACTG<br>Reverse: CCAGGAAGACGGGCTTTTG	64	XM_02108584.7
Mucin2	Forward: GGTCATGCTGGAGCTGGACAGT<br>Reverse: TGCCTCCTCGGGGTCGTCAC	181	XM_021082584.1
Mucin1	Forward: GCTGGTCTGTATTCTGGTCGC<br>Reverse: TCATAGGGGTTCCGTTTGGTA	195	XM_021089730.1
Claudin-1	Forward: TCTTAGTTGCCACAGCATGG<br>Reverse: CCAGTGAAGAGAGCCTGACC<br>Reverse: ACGCCTCCAAGTTACCACTG	106	NM_001244539.1
TNF-α	Forward: TGGCCCCTTGAGCATCA<br>Reverse: CGGGCTTATCTGAGGTTTGAGA	68	NM_214022.1

[1]*β-actin*, beta actin; *ZO-1*, zonula occludens-1, *IL-1β,* interleukin-1β; *MUC1*, mucin-1; *MUC2*, mucin-2; *TNF-α*, tumor necrosis factor-α

Table S2. Intestinal flora RT-PCR primers and probes

Item	Primers/probes	Product size (bp)	Annealing temperature
Total bacteria	Forward: ACTCCTACGGGAGGCAGCAG<br>Reverse: ATTACCGCGGCTGCTGG	61.5	200
Bifidobacterium	Forward: CGCGTCCGGTGTGAAAG<br>Reverse: CTTCCCGATATCTACACATTCCA<br>Probe: ATTCCACCGTTACACCGGGAA	57.9	121
Lactobacillus	Forward: GAGGCAGCAGTAGGGAATCTTC<br>Reverse: CAACAGTTACTCTGACACCCGTTCTTC<br>Probe: AAGAAGGGTTTCGGCTCGTAAAACTCTGTT	53	126
Bacillus	Forward: GCAACGAGCGCAACCCTTGA<br>Reverse: TCATCCCCACCTTCCTCCGGT<br>Probe: CGGTTTGTCACCGGCAGTCACCT	57.9	92
E.coli	Forward: CATGCCGCGTGTATGAAGAA<br>Reverse: CGGGTAACGTCAATGAGCAAA<br>Probe: AGGTATTAACTTTACTCCCTTCCTC	55	96
